# Phenotypical and Genomic Characterization of the Mollusk Pathogen *Francisella halioticida*

**DOI:** 10.1002/mbo3.70172

**Published:** 2025-11-28

**Authors:** Hélène Bouras, Yann Quesnelle, Suzanne Trancart, Didier Goux, Jean‐Louis Blin, Manuel Savary, Maryline Houssin, Céline Zatylny‐Gaudin

**Affiliations:** ^1^ Marine Ecosystems and Organisms Research Laboratory (MERSEA), Esplanade de la Paix University of Caen Normandy Caen France; ^2^ Research Department LABÉO Caen Cedex 4 France; ^3^ CMAbio3 (Centre de Microscopie Appliquée à la Biologie), SF EMerode, Esplanade de la Paix UNICAEN, Normandie Université Caen France; ^4^ CNRS, UMR 6508 CRISMAT, ENSICAEN UNICAEN, Normandie Université Caen France; ^5^ Synergie Mer et Littoral (SMEL) Zone Conchylicole Blainville‐sur‐mer France; ^6^ Comité Régional de Conchyliculture (CRC) Normandie‐Mer du Nord Gouville‐sur‐Mer France

**Keywords:** *Francisella halioticida*, genomics, phenotypical characterization, virulence markers

## Abstract

The emergence and dissemination of aquatic pathogens pose significant risks to farmed species. *Francisella halioticida*, initially reported in abalones and Yesso scallops, was recently isolated from mussels in France, with some isolates showing high virulence. This study aimed to characterize and compare several *F. halioticida* isolates from mussels using phenotypic and genotypic approaches. Phenotypic analysis was performed using growth curves, biochemical profiles (API strips), and morphology assessed by electron microscopy. Genetic analysis has been performed through whole‐genome comparison using classification methods and virulence markers seeking. Phenotypic analyses highlighted similarities among FR22 isolates and notable differences with FR21 and AG1. Notably, AG1 displayed distinct features. Antibiotic resistance profiling revealed the species' capacity to withstand multiple antimicrobial agents with various modes of action. Complete, circular genomes were assembled and compared using targeted and untargeted approaches. These analyses confirmed the affiliation of FR22 isolates with the *F. halioticida* species, while FR21 and AG1 taxonomy need to be further investigated. Virulence factor screening revealed the presence of secretion system components (types I, IV, and VI) in all isolates. A novel variant of the *Francisella* Pathogenicity Island (FPI) was described, shared by all virulent isolates. However, this FPI was absent in the low virulence isolate FR22b. In conclusion, this study discriminates against *F. halioticida* isolates and proposes new hypotheses on their virulence, contributing to improved detection tools and expanding our understanding of this emerging aquatic pathogen.

## Graphical abstract

Phenotypic and genetic characterization of French isolates of *Francisella halioticida* and comparison with Canadian and Japanese strains. The study revealed similarities between FR22s and previous isolates but distinct differences with FR21. A major finding is the absence of the *Francisella* pathogenicity island in the less virulent isolate, FR22b.

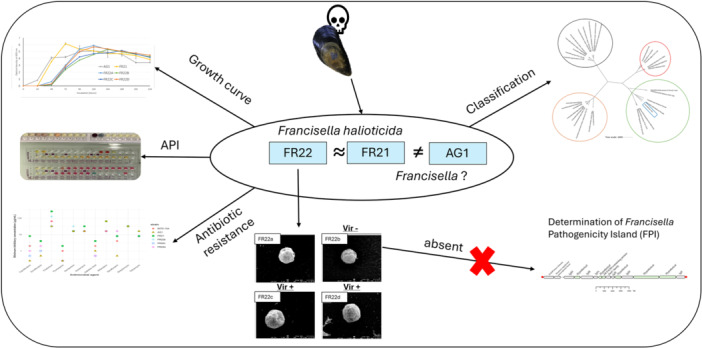

## 1 Introduction

The genus *Francisella* was first described by Dorofeev (1947) with the zoonotic pathogen *Francisella tularensis*, isolated in 1911 (McCoy and Chapin 1912). Due to its transmission via biting or stinging of infected animals or contaminated waters (Francis et al. 1921; Francis and Lake 1921, 1922; Petersen et al. 2009), a reservoir has first been found in ticks where it acts as an endosymbiont (Burgdorfer et al. 1973). With the advances in molecular detection, several environmental research have been going since several decades, highlighting the diversity of the *Francisella* species with both pathogenic and environmental species (Challacombe, Petersen, et al. 2017; Schrallhammer et al. 2011; Sjödin et al. 2014). Phylogenetic analyses reveal four major clades within the *Francisella* genus, mammal pathogens (*F. tularensis*, *Francisella novicida*, *Francisella hispaniensis*, *Fritillaria persica*, and *Francisella opportunistica*), fish pathogens (*Francisella noatunensis*, *Francisella orientalis*, and *Francisella salimarina*), environmental species (including *Francisella halioticida*), and *Francisella*‐associated genera (Challacombe, Pillai, et al. 2017; Kumar et al. 1928; Öhrman et al. 2021).

Compared with environmental species, pathogenic *Francisella* have been the focus of greater scientific interest, leading to a more detailed characterization. Notably, they comprise the only species that are further divided into subspecies: *F. tularensis* and *F. noatunensis*. Into pathogenic species, numerous virulence factors have been highlighted, which can sometimes be found in environmental species, questioning their potential pathogenicity towards unknown host (Meibom and Charbit 2010). In *F. tularensis*, the infection cycle is well known. The bacterium induces endocytosis in the target cell, escape the phagosome before fusion with the lysosome, replicate intracellularly before inducing apoptosis and its release to infect healthy cells. This cycle and the genes associated have been reviewed several times (Asare and Kwaik 2011; Celli and Zahrt 2013; Degabriel et al. 2023; Jones et al. 2012; Meibom and Charbit 2010). A virulence cluster is particularly studied, the *Francisella* Pathogenicity Island (FPI), which encodes for a singular type VI secretion system (T6SS^II^).

The bacterium *F. halioticida* raised interest in molluscan disease during the last decade. First isolated from the giant abalone *Haliotis gigantea* in an affected farm in Japan (Brevik et al. 2011; Kamaishi et al. 2010), it was then found as a pathogen of the Yesso scallop *Mizuhopecten yessoensis* in Japan and Canada (Kawahara et al. 2018; Kawahara et al. 2019; Meyer et al. 2017). *F. halioticida* was also proposed as a potential causative agent of the blue mussel *Mytilus* sp. mortalities in France (Charles et al. 2021; Charles et al. 2020), and our previous studies highlighted the isolation of distinct isolates (Bouras et al. 2023) showing distinct virulence in juvenile and adult mussels (Bouras et al. 2024). In France, mussel mortalities represent the second shellfish farmed. The first mortality events in 2014 have led to significant economic loss with an estimation of 13 million euros (Lupo and Prou 2016). No macroscopic symptoms have been observed in infected mussels in comparison to Yesso scallop presenting abscess on the adductor muscle (Kawahara et al. 2018; Meyer et al. 2017). In the Netherlands, mussel mortalities are also reported since 2016, causing significant economic loss for the farmers (Capelle et al. 2021).

This study aimed to characterize the diverse isolates of *F. halioticida*, pathogens of abalone, Yesso scallop, and mussel across Asia, North America, and Europe, at both the phenotypic and genetic levels. The combined phenotypic and genomic data were used to differentiate between these isolates. Furthermore, full genome sequences were employed to predict and identify virulence markers that could explain the variation in virulence observed specifically among the French FR22 isolates.

## 2 Materials and Methods

### 2.1 Bacterial Isolates and Data Set

This study focuses on the phenotypical and genetical analysis and comparison of *F. halioticida* strains previously isolated from mussels (Bouras et al. 2023). Briefly, FR21, FR22b, and FR22c isolates originate from moribund farmed mussels from local mortality events in Britany and Normandy collected through 2021 and 2022 and stored at −20°C upon harvest to avoid tissue degradation. The isolates FR22d and AG1 were collected in May 2022 from farmed mussels from Agon‐Coutainville, Normandy, outside of mortality events. These mussels were acclimated in 15 L seawater aquariums. Fresh tissue analysis was performed immediately following the death of any mussel. For the isolate FR22a, mussels were collected from wild stocks at Meuvaines, Normandy, and stored at −20°C until further analyses. Bacterial isolation was performed using whole tissues (excluding the digestive glands) for spats (FR21 and FR22b) and gills for adult mussels (FR22a, FR22c, FR22d, and AG1). Tissues were rinsed with artificial sterile seawater and spread onto Modified Eugon Agar (Condalab) plates (MEA) supplemented with 1% hemoglobin (Sigma) (Kamaishi et al. 2005) and complemented with three antibiotics (MEA^3^) to increase the media specificity to *Francisella* species: 50 µg/mL ampicillin, 100 units/mL polymixin B (Soto et al. 2009), and 10 µg/mL erythromycin (Kawahara et al. 2018). Plates were incubated at 15°C or 17°C and monitored daily. Colonies of interest were greyish, convex, and circular with clear margins. Suspected colonies were subcultured on MEA supplemented with 2 mM FeCl_3_ and incubated at 20°C. When subculture on MEA 2 mM FeCl_3_ was unsuccessful, primary colonies were subcultured on MEA 1% hemoglobin and incubated at 20°C.

Identification was performed in subsequent steps. First, subcultures of 24–72 h postincubation were screened through the Matrix Assisted Laser Desorption Ionization‐Time of Flight (MALDI‐ToF) method using the Bruker database 9.0 completed with the *F. halioticida* main spectra profiles produced previously (Bouras et al. 2023). Colonies identified as *F. halioticida* by the MALDI‐ToF and for colonies matching expected morphology, a colony was transferred to 100 µL sterile water, DNA was extracted by boiling lysis (95°C during 10 min) and used for identification through *F. halioticida rpoB* specific Taqman polymerase chain reaction (PCR) following the authors' parameters (Charles et al. 2021). Finally, identification was performed through 16S ribosomal RNA (rRNA) sequencing using conventional primers 27F (5′‐AGA GTT TGA TCM TGG CTC AG) (Lane 1991)/m1492R (5′‐ACC TTG TTA CGA CTT CAC) modified from Lane (1991) and sequenced using the BigDye Terminator v3.1 cycle sequencing kit (Applied Biosystems) following the manufacturer's instructions. All conventional PCR reactions were performed on a T100 Thermal Cycler (Bio‐Rad). PCR amplicons were purified using the ZR DNA sequencing clean‐up kit (Zymo Research) following the manufacturer's instructions, and final products were analyzed on the Genetic Analyzer 3500 (Applied Biosystems).

The previously characterized isolate FR21 as well as the isolate 8472‐13A from yesso scallop in Canada (Kawahara et al. 2018) were used as positive control for phenotypic analysis. All *F. halioticida* isolates were selected for whole‐genome sequencing using hybrid technologies. In addition, the unidentified isolate AG1 (Bouras et al. 2023) was sequenced with the PacBio technology only. The genomes, sequences, and annotations for the strain DSM23729 (CP022132.1) and UTH170823 (AP023082.1) were retrieved from GenBank Genomes.

RefSeq circular genomes of Francisella and related species were retrieved from the National Center for Biotechnology Information. To reduce the data sets, species with more than three genomes represented were submitted to average nucleotide identity (ANI) analysis to confirm their relatedness, and three representative genomes, from different geographic locations, were selected (Table S1). The reference genome was always automatically selected.

### 2.2 Phenotypical Characterization

#### 2.2.1 Growth Curve

Growth curves were performed for all isolates (FR22a, FR22b, FR22d, 8472‐13A, FR21, and AG1) in 250 mL Erlenmeyer flasks filled with 100 mL modified Eugon broth complemented with 4 mM FeCl_3_.

Isolates were previously cultured in this broth. Then, the optical density was normalized at 0.3 OD_600nm_ and diluted 1/50. Growth curve was realized in triplicates, with an incubation at 17°C and agitation at 150 rpm. The growth was followed daily with OD_600nm_ stopped after 3 days in the stationary phase. Statistical analysis was performed using *t* test using an *α* value of 0.05.

#### 2.2.2 Metabolism Study

Biochemical characterization of isolates was performed using API strips, as previously performed for *F. halioticida* isolates (Bouras et al. 2023; Kawahara et al. 2021). Briefly, API strips ZYM, 20E, rapid ID 32 A and 32E (BioMérieux) were inoculated in adequation with the manufacturer's instructions with one modification. To respect osmolarity required by *F. halioticida*, the McFarland solution was realized in physiological water supplemented with 3% NaCl. The strips were incubated at 20°C for 24 h and read as preconized by the manufacturers.

#### 2.2.3 Electron Microscopy

Electron microscopy was performed as in our last study (Bouras et al. 2023). One colony was transferred to 2 or 4 mM FeCl_3_ Modified Eugon Broth (MEB), depending on their requirement, and incubated at 20°C with 180 rpm agitation for 4 days. Cells were pelleted by centrifugation at 3000 *g* for 3 min and rinsed twice in physiological water containing 3% NaCl. Pellets were fixed with 2.5% glutaraldehyde in cacodylate buffer (0.2 M, pH 7.4 with 7% sucrose) at 4°C overnight and rinsed with cacodylate buffer. Cells were postfixed for 1.5 h with 2% osmium tetroxide in cacodylate buffer (0.2 M, pH 7.4 with 7% sucrose) and rinsed with the same buffer.

For transmission electron microscopy (TEM), cells were pelleted in 1.5% low‐melting‐point agar at 40°C. The cells were then dehydrated in progressive ethanol baths (70%–100%) and embedded in Embed 812 resin before being polymerized at 60°C for 48 h. Ultrathin sections were produced and contrasted with uranyl acetate and lead citrate. Observations were made with a transmission electron microscope (JEOL 1011), and images were taken with an ORIUS 200 Camera using digital Micrograph software.

For scanning electron microscopy (SEM), cells were sedimented on Thermanox coverslip coated with poly‐l‐lysine. The cells were then dehydrated in progressive ethanol baths (30%–100%) and critical point dried (CPD 030 LEICA Microsystem). Cells were sputtered with platinum and observed with a scanning electron microscope (JEOL 7200 F). Samples were prepared and analyzed at the Electron Microscopy Center of the University of Caen (CMAbio3) for TEM and at CRISMAT for SEM.

#### 2.2.4 Antibiotic Resistance

Resistance profiles of the isolates were performed in the scope of resistance research to increase knowledge and help improve isolation media. Antimicrobial resistance was assessed with the µ‐dilution broth method for the isolates FR22b, FR22c, FR22d, 8472‐13A, FR21, and AG1. Isolated were cultured in MEA supplemented with 4 mM FeCl_3_ and incubated at 17°C for 72 h before the analysis. Selected antibiotics (Table S2) were plated as the conventional method preconize, using one column for positive control (bacteria without antibiotics) and one line for negative control (antibiotics without bacteria). Bacteria were prepared in physiological water with 3% NaCl at 0.5 McFarland and plated accordingly. All tests were performed in triplicates. Plates were incubated at 17°C and read at 3, 6, and 10 days. The minimum inhibition concentration (MIC) was determined as the first concentration without bacterial growth. Positive controls have been performed with *Escherichia coli* ATCC 25922 and *Staphylococcus aureus* ATCC 29213 grown at 37°C in their respective media to validate the concentration of the antibiotics solutions for which EUropean Committee of Antimicrobial Sensibility Testing (EUCAST) breakpoints were available (European Committee for Antimicrobial Susceptibility Testing of the European Society of Clinical Microbiology 2024). Statistical analysis was performed using *t* test using an *α* value of 0.05.

### 2.3 Whole‐Genome Sequencing

#### 2.3.1 Strains

The strains were cultured in 2 mM FeCl_3_ MEB at 20°C with 190 rpm agitation for 4 days and 2 mL of broth culture was used to extract DNA. In short, cells were pelleted and suspended in 100 µL molecular grade water before DNA extraction using the QIAmp DNA Mini Kit (Qiagen) following the manufacturer's instructions with the exception of the elution performed in the elution buffer (EB) (Qiagen). The quality of DNA extracts was checked by Nanodrop 2000 (Thermo Scientific), and the quantification was performed with Qubit 1X dsDNA Assay kit on Qubit Fluorimeter 4 (Invitrogen). Samples were submitted to short‐read (Illumina) and long‐read (PacBio Sequel II) sequencing. Illumina libraries were prepared using the Nextera CD 24 DNA Library Preparation Kit (Illumina), verified with the Fragment analyzer (Agilent) and sequenced on the iSeq. 100 sequencer (Illumina) at LABEO laboratory facilities. Depth of sequencing was set at 40×. PacBio Sequel II sequencing was performed by the Gentyane INRAE platform (Clermont‐Ferrand, France).

#### 2.3.2 Assembly

For Illumina sequencing reads, the removal of adapters and trimming of low bases quality was performed with Trimmomatic v.0.39 (Bolger et al. 2014) (ILLUMINACLIP:NexteraPE.PE.fa:2:15:1, LEADING:3 TRAILING:3 SLIDINGWINDOWN:4:20 MINLEN:30). Quality of the reads was performed with FastQC (Andrews 2010) and MultiQC (Ewels et al. 2016). Raw reads obtained from PacBio were preprocessed with Filtlong v.2.0.1 without external reference (min_length 1000 keep_percent 90) (R. Wick 2021). Genomes were assembled with Unicycler v0.5.0 using default parameters (R. R. Wick et al. 2017). Chromosomal sequence and potential plasmid sequences were split, and annotation was performed using the prokaryotic genome annotation pipeline (PGAP) (Tatusova et al. 2016). Assessment of the quality of assembly was performed with QUAST v.5.2.0 (Gurevich et al. 2013), and completeness of genome assembly and annotation was performed with BUSCO v.5.4.2 (Seppey et al. 2019).

### 2.4 Analysis

#### 2.4.1 Identification

To perform a complete identification and assess the placement of our strains in the *Francisella* and related genera, several methods were combined. An Average Nucleotide Identity (ANI) analysis was realized with ANIclustermap v1.3.0 (Shimoyama 2022). A digital DNA–DNA hybridization (dDDH) was performed using the Genome‐to‐Genome Distance Calculator 3.0 (Meier‐Kolthoff et al. 2013; Meier‐Kolthoff et al. 2021) available on DSMZ's web server (Meier‐Kolthoff et al. 2014). Finally, the placement of the different isolates was also assessed by using the pangenome approach. The software PanACoTA (Perrin and Rocha 2021) available on the PanExplorer web server (Dereeper et al. 2022) was used with default parameters. For all genomes, the GenBank format was used as entry, either with accession number (Table S1) or PGAP output for the isolates sequenced in this study.

#### 2.4.2 Chromosomic Arrangement

The genomes of the isolates, which were confirmed as *F. halioticida*, were aligned and visualized using the software ProgressiveMauve 2015‐02‐1 (Darling et al. 2010).

#### 2.4.3 Identification of Genes of Interest

In this study, the genes of interest were the main characterized virulence genes in *Francisella* species. The virulence genes are well known in *Francisella* species pathogens of mammals or fish (Degabriel et al. 2023). The first goal was to identify and determine if an FPI was present in *F. halioticida*, which was performed by the localization of the genes intracellular growth locus A and B (*iglA* and *iglB*) previously identified in *F. halioticida* (Kawahara et al. 2021) and finding homologous sequences of other FPI genes in the surroundings. In bivalves' pathogens, metallopeptidases are genes of virulence of interest. Using the MEROPS output of METABOLIC‐G v4.0 run with default parameters (Zhou et al. 2022) associated with primary identification by PGAP of metallopeptidases, a comparison of the presence/absence was performed.

### 2.5 Validation of the Presence of FPI

To validate the presence or absence of the FPI in the isolates, a conventional PCR was designed (*iglA*_conv_F, 5′‐ATTCCTAAATCGCGCATCACAATTAC‐3′; *iglA*_conv_R, 5′‐AAGTTGGTATGATTCAGTAGCATTAAGC‐3′) to produce an amplicon of 480 bp. PCR amplification was performed as in our previous study (Bouras et al. 2023). The reaction mixture consisted of 1X Premix Ex‐Taq (Takara), 0.4 µM of each primer, 2 µL of DNA or water (negative control) and completed with molecular grade water up to 25 µL. The thermal cycling conditions were the following: initial denaturation at 95°C for 10 s followed by 40 cycles at 95°C for 10 s, 55°C for 30 s, 72°C for 40 s, and a final extension was performed at 72°C for 3 min. PCR products were visualized by migration on QIAxcel Advanced System (Qiagen) and quantified by Qubit 1X dsDNA Assay kit on Qubit Fluorimeter 4 (Invitrogen). Amplicon concentrations were adjusted to 8–10 ng/µL and sequencing reactions were performed using the BigDye Terminator v3.1 cycle sequencing kit (Applied Biosystems) following the manufacturer's instructions. All conventional PCR reactions were performed on a T100 Thermal Cycler (Bio‐Rad). Amplicons were visualized on the QIAxcel Advanced System (Qiagen).

To control the presence of strains exhibiting the FPI, a duplex TaqMan PCR was designed on the gene Peptide Chain Factor Release RF2 (*prfB*) aiming to detect the presence of *F. halioticida* strains as well as the virulence gene *iglA* (Table 1). Mussels were harvested during mortality events (list in Supporting Information Data, Table S3), gills were taken and gathered to produce pools of four animals per site. These samples were submitted to PCR.

**Table 1:** Polymerase chain reaction primers and probe for the detection of *Francisella halioticida* and the virulence gene in field samples.prfB : Peptide chain release factor RF2. iglA : intracellular growth locus A.

Gene	Name	Primer sequence (5′ → 3′)
*prfB* (109 bp)	Fh_*prfB*_F	CTTATGCAAGARATTGCTAGA
	Fh_*prfB*_R	CAAGATAGGCATTGTTAGTAT
	Fh_*prfB*_Probe_halioticida	TexasRed‐ACGACTAAATTCAAGTTTTTCTATTTCACT‐BHQ2
*iglA* (124 bp)	Fh_*iglA*_F	ATTCCTAAATCGCGCATCA
	Fh_*iglA*_R	TTCTTTCCTCTGAATTTCCTA
	Fh_*iglA*_Probe	FAM‐TAGTTCCATCAACTTCCATATCATAAGTA‐BHQ1

The reaction mixture consisted of 1X Premix 1X Premix Ex‐Taq (Takara), 0.2 µM of forward primer, 0.4 µM of reverse primer, 0.2 µM of probe, 2 µL of DNA or water (negative control), and completed with molecular grade water up to 25 µL. Thermal cycling started with a fast enzyme activation of 95°C for 10 s followed by 45 cycles of denaturation at 95°C for 5 s, hybridization at 53°C for 30 s, and elongation at 72°C for 30 s. Amplification was performed on a Quant Studio 5 (Applied Biosystems, Life Technologies) thermocycler and analyzed with the Quant Studio software.

## 3 Results and Discussion

### 3.1 Bacterial Phenotypical Characterization

**Figure 1:**
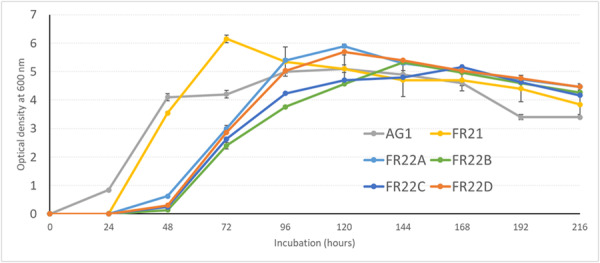
Growth curve of *Francisella* spp. obtained from mussels on the French coast. AG1, uncharacterized species of *Francisella*; FRxx, *Francisella halioticida*.

Two distinct growth patterns are observed among the mussel *Francisella* isolates (Figure 1) (*p* < 0.05). FR21 and AG1 exhibit a shorter lag phase, while isolates FR22a, b, c, and d showed a longer lag phase of approximately 48 h. In general, the isolates exhibit a long exponential phase between 48 and 72 h. For FR22 isolates, while FR22a and FR22d rapidly reach a higher optical density followed by a senescence phase, isolates FR22b and FR22c enter a stationary phase with a slight increase in optical density for 48 h. No significant differences have been observed at the stationary or senescence phase.

In a general manner, the isolates did show a similarity (see Table S4 for full table) but differences allowed the differentiation between isolates (Table 2). The first difference concerns esterase (C4) and esterase lipase (C8) activities, which are lower in FR22 isolates than in isolates 8472‐13A and FR21. Other differences were observed for the aminopeptidases used to degrade phenylalanine, leucine, arginine, and serine. The differences are mainly due to lower leucine aminopeptidase activity and higher arginine aminopeptidase activity in FR22d compared with the other FR22 isolates. Isolate FR22c showed higher serine aminopeptidase activity than other FR22s.

**Table 2:** Biochemical characteristics of *Francisella halioticida* and *Francisella* sp. AG1 isolated from mussels.

	8472‐13A	FR21	FR22a	FR22b	FR22c	FR22d	AG1
Acetoin production	−	+	−	−	−	−	−
Mannose fermentation	−	+	−	−	−	−	W
Esterase (C4)/Esterase lipase (C8)	+	+	W	W	W	W	N/R
Leucine aminopeptidase	+	+	+	+	+	W	+
Naphtol‐AS‐bi‐phosphohydrolase	+	W	W	W	W	W	N/R
Phenylalanine aminopeptidase	W	+	−	−	−	−	−
Serine aminopeptidase	+	+	−	W	+	W	+
Tyrosine aminopeptidase	−	+	−	−	−	W	W
β‐Glucuronidase	−	W	−	−	−	−	−
Prolyl aminopeptidase	+	+	+	+	+	+	−
Pyroglutamic acid aminopeptidase	+	+	+	+	+	+	−
Arginine aminopeptidase	+	+	−	−	−	W	−
Indoxyl phosphate	W	W	W	W	W	W	+

*Note:* The biochemical capacity of each isolate was assessed by API strips ZYM, rapid ID 32A, and rapid ID 32E and 20E. The isolates 8472‐13A and FR21, previously characterized (Bouras et al. 2023), were used as positive controls. −, negative; +, positive; N/R, not realized; W, weak signal. Due to the nature of these colorimetric tests, a weak signal was characterized as medium changes between the negative and positive expected results.

**Figure 2:**
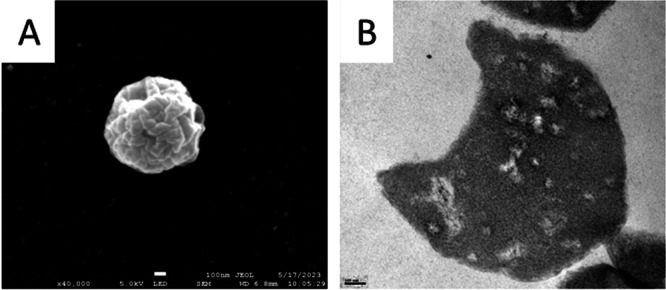
Electron microscopy of *Francisella halioticida* FR22a cultured in modified Eugon Broth. (A) Scanning electron microscopy, scale = 100 nm. (B) Transmission electron microscopy, scale = 100 nm.

In electronic microscopy visualization, high similarity was observed in isolates belonging to the strain type FR22. They all present a cocci shape with an invagination in one pole, observed both in scanning and transmission microscopy. The example for the isolate FR22a is displayed (Figure 2), and captions for FR22b, FR22c, and FR22d are available in Supporting Information Data (Figures S1 and S2).

### 3.2 Antibiotic Resistance

**Figure 3:**
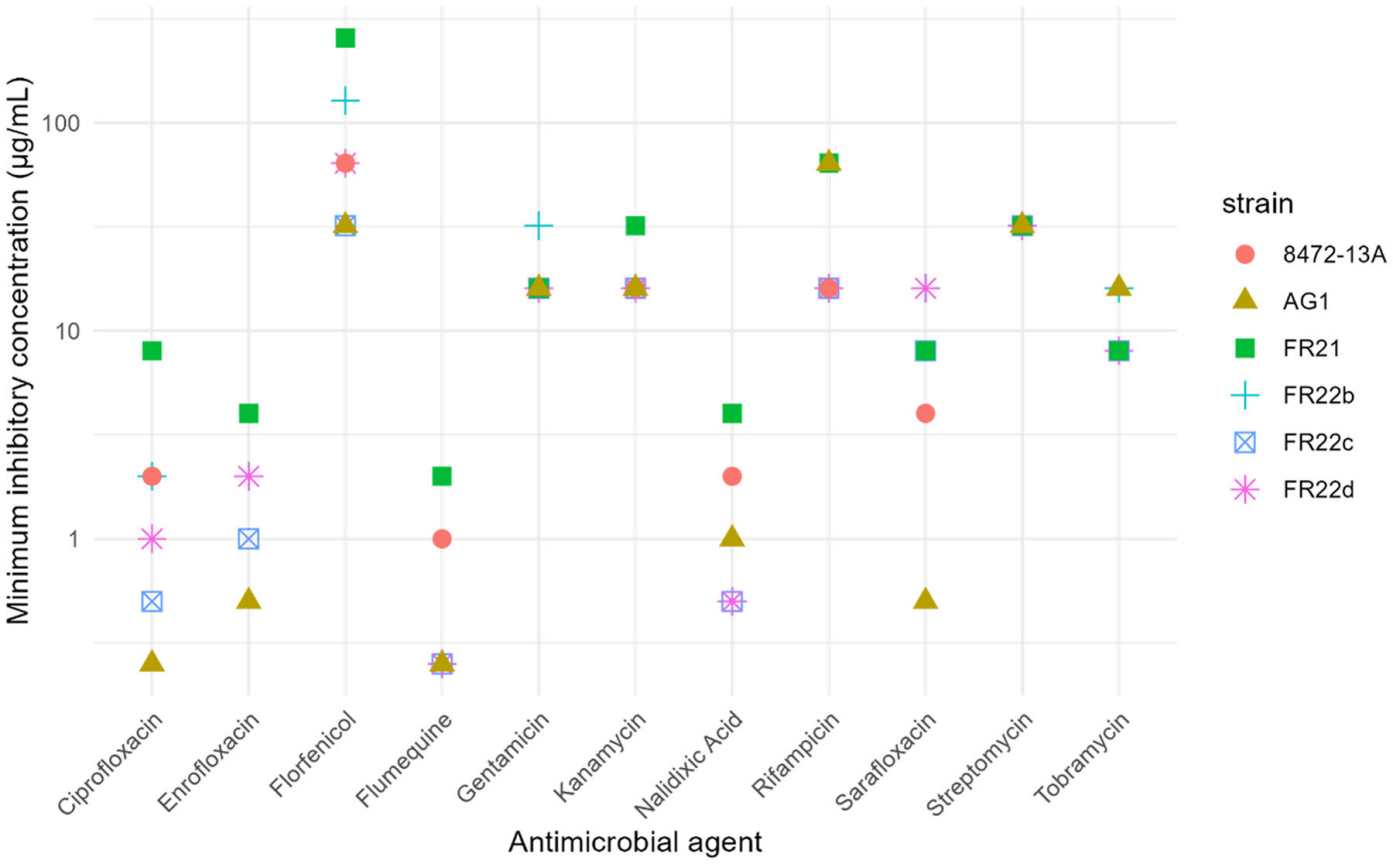
Minimum inhibitory concentration (µg/mL) of 11 antimicrobial agents from four distinct families in *Francisella halioticida* isolates and *Francisella* sp. AG1.

For each isolate–antibiotic combination, the triplicates yielded identical results. All isolates showed a high resistance to several antimicrobial agents, resisting the highest concentration for amoxicillin, ampicillin, penicillin (> 512 µg/mL), cefuroxime (> 512 µg/mL), oxytetracycline (> 8 µg/mL), doxycycline (> 16 µg/mL), erythromycin (> 128 µg/mL), polymixin B (> 128 µg/mL), sulphamethoxazole (> 512 µg/mL), and trimethoprim (> 32 µg/mL). As fungal invasion was observed during the isolation attempts, an antifungal study was carried out. All isolates were again resistant to the highest concentration tested for amphotericin B (> 32 µg/mL), cycloheximide (> 64 µg/mL), and natamycin (> 64 µg/mL). MIC values for all isolates were full resistance to the tested concentration, which was not met concerning molecules belonging to the aminoglycoside/aminoglycoside, phenicol, rifamycin, and fluoroquinolone/quinolone families (Figure 3).

In the case of aminoglycoside/aminoglycoside representatives, it appears that isolates are sensitive to MICs of between 8 and 32 µg/mL, depending on the isolate and the antibiotic tested. Florfenicol showed a significant difference (*p* < 0.05) within the MIC values between the isolates FR22c and AG1 (32 µg/mL) compared with the other isolates, FR21 (> 256 µg/mL) and FR22b (> 128 µg/mL). The isolates FR22d and 8472‐13A showed an intermediate resistance with a MIC value of 64 µg/mL. MICs also varied for fluoroquinolones and quinolones. For all five molecules tested, isolate AG1 appeared to be the most sensitive and FR21 the most resistant (*p* < 0.05). Although a higher concentration of sarafloxacin (16 µg/mL) had to be used for isolates FR22b and FR22d. A distinct pattern is also visible for the rifampicin between isolates AG1 and FR21 (64 µg/mL) and the other isolates FR22 and 8472‐13A (16 µg/mL) (*p* < 0.05).

### 3.3 Assembly

The hybrid assembly as well as the long‐read assembly of AG1 produced a single circular chromosome for each isolate, each having a BUSCO score between 98% and 100% of completeness on the Thiotrichales database (Table 3). One to five plasmids were obtained for each strain, either directly from Unicycler assembly or via a novel specialized assembly using metaplasmidSPAdes (Table 1). While FR21 seemed to present a unique plasmid (130,507 bp), the AG1 isolate did seem to present two plasmids (74,083 and 39,551 bp).

**Table 3:** Assembly characteristics of the genomes of *Francisella halioticida* FR22a, FR22b, FR22c, FR22d, 8472‐13A, FR21, and *Francisella* sp. AG1.

	FR21	FR22a	FR22b	FR22c	FR22d	8472‐13A	AG1
Chromosome	1	1	1	1	1	1	1
Size (bp)	2,287,417	217,480	2,141,138	2,160,324	2,171,110	2,169,425	2,083,160
Circular Plasmids	1	3	3	3	5	5	2
Non‐assembled reads	No	No	Yes	Yes	No	No	Yes
CDS	2141	2241	2208	2219	2241	2248	1984
rRNA	4/3/3	4/3/3	4/3/3	4/3/3	4/3/3	4/3/3	4/3/3
tRNA	38	39	39	39	39	40	38
ncRNA	4	4	4	4	4	4	4
Pseudogenes (F/I/S/M)	45 (24/25/11/13)	119 (46/71/36/29)	117 (46/69/36/29)	119 (49/68/37/30)	119 (48/65/37/25)	110 (45/64/39/32)	97 (49/59/10/19)
CheckM score	98.55/4.01	96.59/2.87	96.59/2.87	96.17/2.87	96.48/2.87	97.01/2.36	94.48/2.24

*Note:* The information concerning the number of coding DNA sequences (CDS), ribosomal RNA (rRNA), transfer RNA (tRNA), noncoding RNA (ncRNA), pseudogenes (Frameshift/Incomplete/Stop codons/Multiple causes), and checkM score was generated by the Prokaryotic Genome Annotation Pipeline (PGAP).

The Canadian isolate 8472‐13A does possess a plasmid of distinct size compared with those from French isolates. The isolate 8472‐13A did present a plasmid of 56,863 bp, two around 20 kbp (22,066 and 21,410 bp), and two small (4017 and 3003 bp). The French isolates did present similar plasmid size, with one comprised between 40,609 and 46,511 bp and two small plasmids of nearly 5 and 3 kbp. No plasmid was associated with the reference strain DSM23729 (Uneklint et al. 2020), and two were described for UTH17823 (Kawahara et al. 2021).

### 3.4 Identification

The unidentified species AG1 at the 16S rRNA level did not show any relatedness with any included species, which is not surprising as databases are joined. However, this analysis allowed the classification of this isolate within the Francisellaceae family. Within the family, any two distinct species show an ANI score ranging from 70% to 80%. The input of *Fangia hongkongensis*, which belongs to the same order, Thiotrichales, had no pertinent similarity and was classified with an ANI score of 0. Together, this analysis seemed to indicate that this isolate belongs to a novel species, but it is impossible to classify its belonging to the genus *Francisella* or any related genus.

As with several genera, *Francisella* species are sometimes classified into subspecies, and the criteria might depend. In this study, the isolate FR22 does seems from the primary observation belong to the species *F. halioticida*, while FR21 did present slight differences (Bouras et al. 2023). Along with, we do have another isolate for which no identification could be obtained from the 16S rRNA. The ANI is a popular method to estimate relatedness between isolates or species. Using isolates from several species belonging or related to *Francisella* allows one to narrow the prediction for these isolates (Figure S1). First, the isolates FR22 do present a high similarity (> 99%) within themselves but also with the other isolates, DSM23729, 8472‐13A, and UTH170823.

While the highest similarity with the isolate FR21 is the isolates *F. halioticida*, the ANI score remained lower with 96.7%–96.8%, which remained higher than the conventional threshold of 95%. However, if we look at this value within the *Francisella* genus, it appears that the ANI score between *F. tularensis* subsp. *tularensis* and *F. tularensis* subsp*. holarctica* was 99.1%. In the same manner, the species *F. novicida*, which was debated in the form of subspecies or distinct species present an ANI score of 97.5%–97.9% with *F. tularensis tularensis*. However, this index cannot be used alone; other tools must be added to establish a novel classification.

The dDDH value is another variable used to distinguish between two species. Among *F. halioticida* isolates, a high degree of similarity is observed, with an estimated hybridization of 99.3%–99.4% between the FR22 isolates and 98.6%–98.8% with the Japanese and Canadian strains. It is important to note that the Canadian strain, 8472‐13A possesses a dDDH value of 98.8% with UTH170823 but 99.9% with DSM23729. Once again, the FR21 isolate remained singular, with the *F. halioticida* presenting dDDH values ranging from 79.3% to 79.6%. For information, the closest species to *F. halioticida* based on the dDDH value is *F*. uliginis at 30.5%–30.6%.

Among the other isolates tested (Table S1), the dDDH between distinct species ranged between 21% and 22.5% within the *Francisella* genus and less than 21% between *Francisella* and related genus. In contrast to the ANI analysis, *Fangia hongkongensis* showed the best dDDH value with 27.5% with *F. halioticida*. However, the %GC allowed better discrimination, with 0.3%–1.5% between *Francisella* species and 6.75% between *F. halioticida* and *Fangia hongkongensis*. Once again, the isolate AG1 remains unrelated to any known species when analyzed with this methodology. Main values are located between 20% and 21% whether the species belongs to *Francisella* or a related genus. However, it is important to note that a weak comparative score was obtained against the *Pseudofrancisella* genus (18.8%–19.1%).

In this study, the pangenome was used as a comparison tool to produce a phylogenetic tree aiming to place the different isolates. Even if the other characteristics were not analyzed, the size of the core genome was considered for several conditions. First, a core genome of 508 genes, completed with 3860 genes belonging to the shell genome and 3572 to the cloud genome, was observed over the whole *Francisella* genus. When selecting only *F. halioticida* isolates, a core genome of 1612 genes was observed, representing 73% of the pangenome. The integration of FR21 in this pangenome drastically reduces the core genome to 1335 genes (47.7% of the pangenome). When adding the closest species, *F. uliginis* instead of FR21, the number of genes in the core genome dropped to 997 and got back to 1193 when both were added. A similar study was tested with *F. tularensis* subspecies using the following isolates for *F. tularensis* subsp. *tularensis* (GCA_000833535.1, GCA_000833475.1, GCA_001262115.1, GCA_001267475.1, GCA_001011135.1, GCA_000009325.1, and GCA_016604735.1). The pangenome was realized once with *F. tularensis* subsp. *mediasiatica* 2023/540 and once with *F. orientalis* F1. The core genome represented 58.5% of the pangenome with *F. tularensis* subsp. *tularensis* alone, 58.5% with *F. tularensis* subsp. *mediasiatica* and dropped to 26.6% with *F. orientalis*.

**Figure 4:**
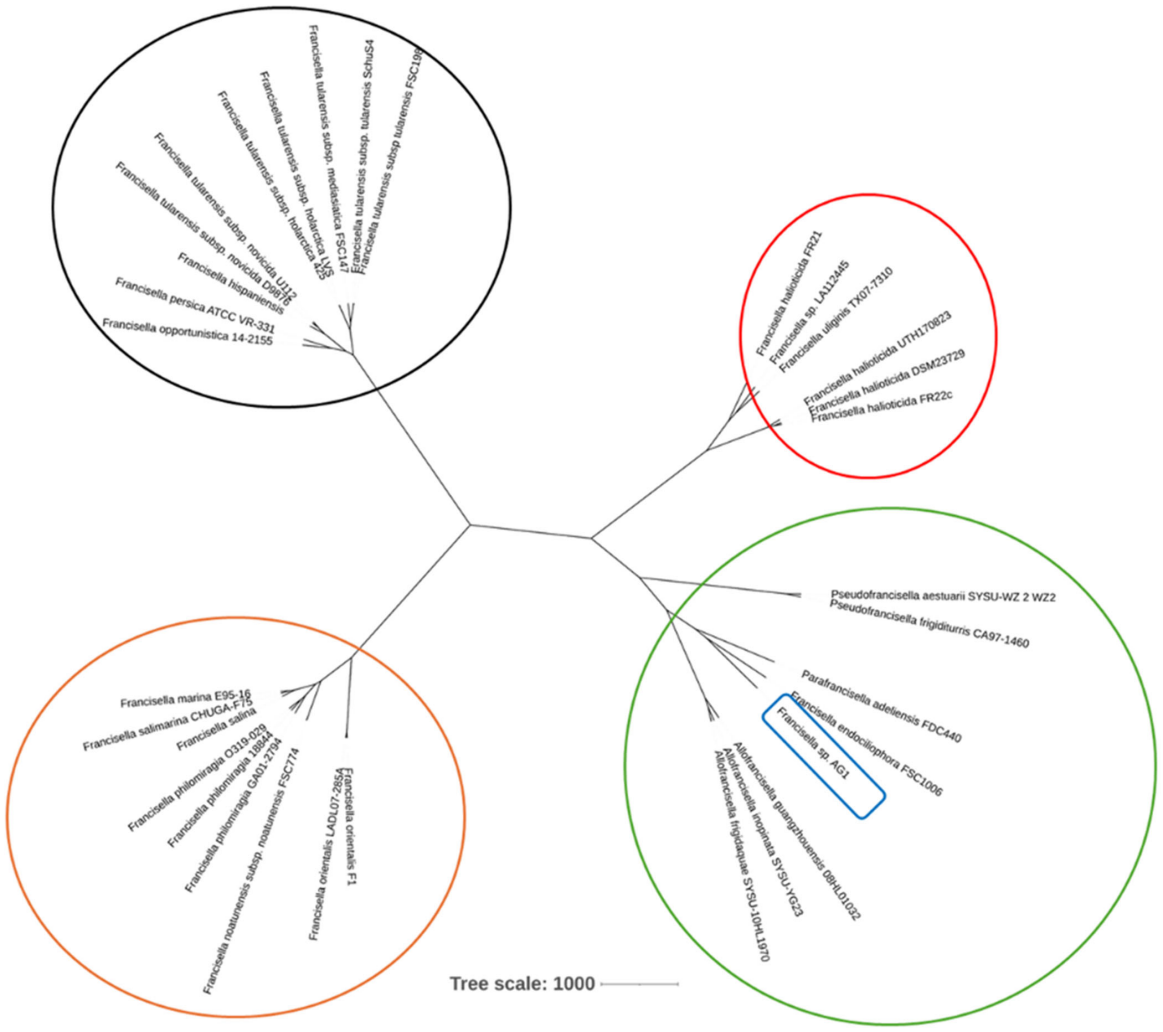
Phylogenetic tree computed from the pangenome of isolates belonging to species of the genus *Francisella* or related. The pangenome was produced with the software PanACoTA on the web server PanExplorer. Black circle (left, up), mammal pathogens, including *Francisella tularensis*; orange circle (left, down), fish pathogens, including *F. orientialis* and *Francisella noatunensis*; red circle (right, up), environmental and mollusks pathogens species, including *Francisella halioticida*; green circle (right, down), genera associated with the genus *Francisella*.

The phylogenetic tree constructed from the pangenome replaces the four clades as described previously (Figure 4). With the clade containing *F. halioticida*, FR21 is presented on a different branch than *F. halioticida*, rather gathering with *Francisella* sp. LA112445 or *F. uliginis*. The isolate AG1 is clustered with the clade containing the related genera to *Francisella*, alongside the species *Francisella endociliophora*. However, neither of these two isolates seemed to be related to previously described species or genera.

### 3.5 Francisella halioticida

#### 3.5.1 Chromosomal Organization

**Figure 5:**
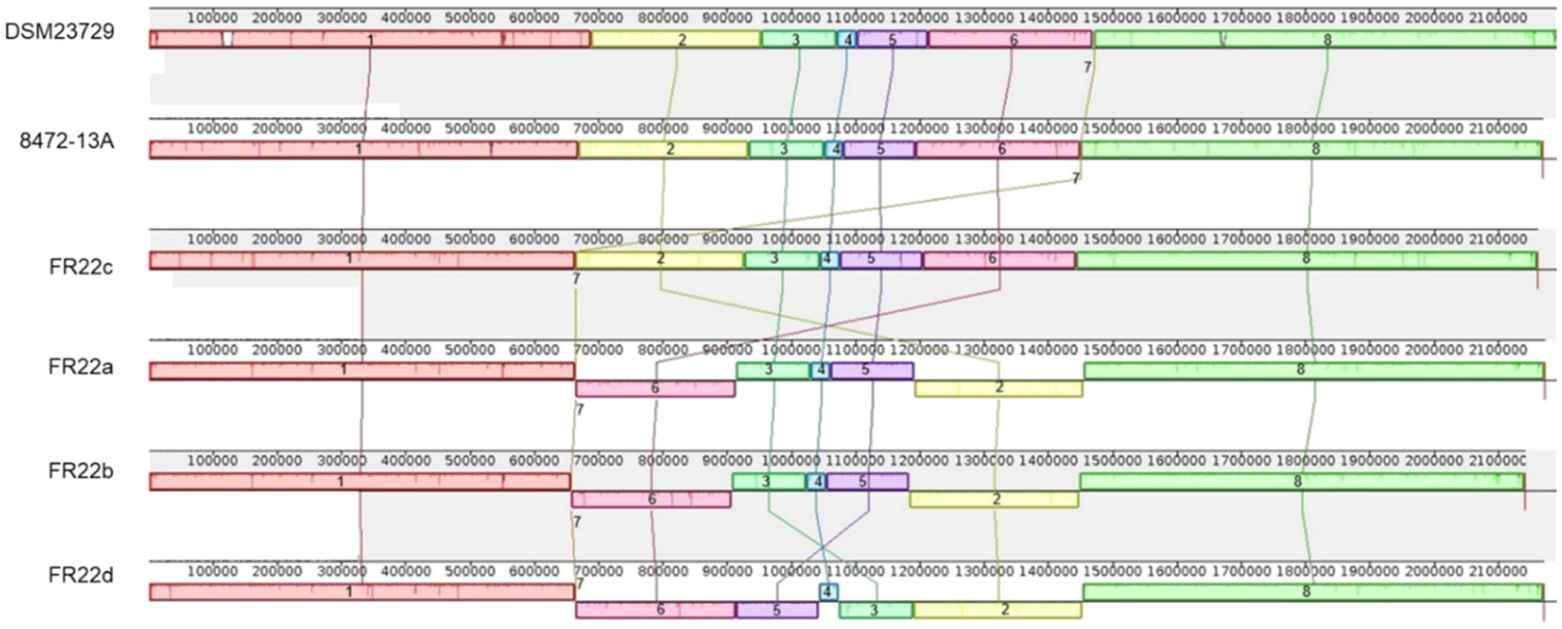
Multiple alignment of the genomes of six isolates belonging to the species *Francisella halioticida*. This alignment highlights chromosomal reorganization. The blocks have been numbered according to the reference isolate, DSM23729.

The French isolate strictly identified as *F. halioticida* FR22a, FR22b, FR22c, FR22d, and the Canadian isolate 8472‐13A were aligned with the reference strain DSM23729 (Figure 5). Graphical visualization shows that eight major blocks were retained between the six isolates. While differences in organization were observed between the different FR22 isolates, isolates DSM23729, 8472‐13A, and FR22c, isolated from animals on three different continents, showed a very similar genome organization. The only difference observed between FR22c and DSM23729 and 8472‐13A is the difference in the location of block 7 in the second position in the FR22c genome (between 60,000 and 70,000 bp), whereas it was present in position 7 in the DSM23729 and 8472‐13A genomes (between 140,000 and 150,000 bp). This block is conserved in terms of position in the four FR22 isolates. A reorganization is visible with a permutation of blocks 6 and 2 with a strand inversion (+ for DSM23729, 8472‐13A, and FR22c vs. − for FR22a, FR22b, and FR22d). While the location of blocks 3–5 is conserved for the FR22a and FR22b genomes, a new inversion between blocks 3 and 5 is observed in the FR22d genome. Each block is flanked by genes encoding IS3 family transposases, with the exception of block 7, which is composed entirely of genes encoding transposases. The reduced size of FR22b can also be linked to block 8.

#### 3.5.2 *Francisella* Pathogenicity Island

**Figure 6:**
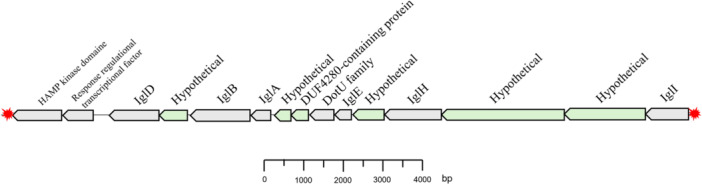
Determination of the “*Francisella* Pathogenicity Island” (FPI) in *Francisella halioticida*. Red stars, transposase IS3; gray blocks, identified with BLAST; green blocks, hypothetical.

Within the genome of *F. halioticida* isolates, a cluster containing both the previously described virulence gene *iglA* and *iglB* genes can be associated with this FPI island (Figure 6). This cluster was found in every genome except in the isolate FR22b.

This cluster is present on the complementary strand flanked by genes encoding IS3 transposases, but also elements annotated as the remnant of a transposase action. This gene cluster starts at around 160,000 bp when the chromosome starts with *dnaA*, corresponding to block 8 of the multiple genome alignments (Figure 5). This cluster has a size of 16,648 bp in all isolates except 8472‐13A (16,015 bp), which lacks the gene coding for a histidine kinase sensor containing a HAMP domain at the 3′ end (Histidine kinases – Adenylate cyclases – Methyl‐accepting chemotaxis proteins – Phosphatases). It includes the genes required for intracellular growth (“intracellular growth locus”) *iglA*, *iglB*, *iglD*, *iglE*, *iglI*, and *iglH*. In isolate 8472‐13A, the difference in cluster size is due to a truncated FPI at the 3′ end. This gene is followed in all isolates by a transcriptional regulatory factor. This cluster is arranged identically to all isolates and some low polymorphisms can be detected in some genes, such as IglA, IglD, IglH or the histidine kinase sensor.

In the case of FR21, the cluster illustrating FPI is clearly present, with high conservation of the proteins encoded by the genes presented above (Figure 6). An identity of between 95% and 98.3% was observed for all the amino acid sequences with the exception of the hypothetical protein located before IglH (94.7%) and IglI (91.8%). Furthermore, the genes surrounding this cluster code for transposases of the IS630 family and not IS3. Another major difference is the number of genes between these transposases. Whereas stricts *F. halioticida* contain only this FPI‐type cluster in their genome, the FR21 genome has the *ompH* gene as well as a gene coding for a hypothetical protein and a gene containing the DUF378 domain upstream of the cluster. Downstream, the gene for a phosphopentomutase and a number of genes involved in sugar or acyl‐CoA metabolism were observed. These additional genes increase the total number of genes by 30. For strict *F. halioticida* isolates, these genes were found outside this chromosomal section.

For AG1, a pseudo‐FPI can be determined by locating the *iglA, B, D*, and *E* genes. It could be located arbitrarily between position 64,651 bp, beginning with *iglE* and position 89131 corresponding to the response regulator transcription factor gene, with reference to the *F. halioticida* cluster. This predictive IPF is composed of the *iglA, B, D, E, I*, and *H* genes, as well as the genes encoding the d*otU* T4/6SS proteins and the histidine kinase sensor containing a HAMP domain. In addition, genes not observed in *F. halioticida* or FR21 such as *tyrA*, *dtd*, or *kdpA* (in pseudogene form) are present.

#### 3.5.3 Other Secretion Systems

Apart from the FPI described, other genes associated with secretion systems are found in the chromosome of *F. halioticida* and isolate AG1. First, the type I secretion system (T1SS), which is the simplest TSS present in all gram‐negative bacteria, was found. This system consists of a pore on the outer membrane (TolC), a specialized ABC transporter of α‐hemolysin with ATP binding located on the inner membrane (HlyB) and a membrane fusion protein (HlyD) (Tseng et al. 2009). In *F. halioticida*, three genes from the tolC family are found, two of which are perfectly identical between *F. halioticida* isolates FR22a, b, c, d, 8472‐13A, UTH170823, and DSM23729. Two genes from the *hlyD* family are also present, one of which is only found in *F. halioticida* (100% identity) with the exception of isolate UTH170823. The second is present in all *F. halioticida* strains and is also found in *F. hispaniensis*, *F. tularensis* subsp. *novicida*, *F. opportunistica* and *Allofrancisella guangzhouensis*. No gene has been directly identified as *hlyB*, but several ABC transporters have been found in *F. halioticida* and could be used in this secretion system.

Two members of the type IV secretion system (T4SS), *virB9* and *virB10*, were detected in isolate UTH170823. These sequences are located between two transposition elements at positions 1,997,677–1,999,545, that is, in block 5 (Figure 5). Searches for these DNA sequences using the “megablast” algorithm yielded no results. The protein sequences have no correspondence with other *Francisella* species, some of which do contain these genes. When compared with *F. philomiragia* or *F. tularensis* at the protein level, only 20% identity was observed between the sequences of our isolates and these two species, whereas 99.18% identity was observed between them.

With regard to the T6SS, two associated genes were found outside the FPI, *anmK* and *clpB* in *F. halioticida*. They are located away from this FPI at positions 1,081,504, 1,406,051 and 2,015,686, respectively. The *vgrG* gene, neither annotated nor identified in *F. halioticida* and FR21, is directly annotated in AG1 as VgrG/Pvc8.

The isolate AG1 also presents several sequences of phage origin, encoding for baseplate, flagella, capsid, and tube.

#### 3.5.4 Metallopeptidase

Given their importance in the virulence of certain bacterial species in bivalves, a search for metallopeptidases was carried out in *F. halioticida*. Seven metallopeptidases were directly identified by annotation of the *F. halioticida* genome. The genes encoding the membrane proteins FtsH and HtpX, the cytoplasmic protein SprTe and a type II member of the CAAX Protease and Bacteriocin‐Processing Enzyme (CPBP) family were identified. The peptidase “Type II CAAX endopeptidase family protein/CPBP family intramembrane glutamic endopeptidase” from the MEROPS G05 family appears to be present only in FR22 within the *F. halioticida* species. When investigating, it appears that truncated version presenting the N‐terminal was present in isolates UTH170823 and DSM23729.

The M3, M14, and M20 families were directly identified as such. A search of the MEROPS database identified new metallopeptidases belonging to families M15, M16, M19, M24, M32, M38, and M50.

In total, this study highlights 14 potential metallopeptidases in *F. halioticida*, two of which are specific to *Francisella*. No similarity was observed with metallopeptidases found in bivalve pathogens from the *Vibrio* genus. Isolate AG1 does not appear to possess genes encoding the CPBP family protein, but conservation of other metallopeptidases was observed.

## 4 Discussion

The development of bacterial diseases in open‐sea shellfish farming presents a major challenge, as curative treatments are limited or impossible to apply. In France, mussel farming relies mainly on wild‐caught spat, collected from the natural hybrid zone between *Mytilus edulis* and *Mytilus galloprovincialis*, thereby precluding genetic selection for disease resistance. While hybrid mussel may display variable resistance to disease, mortality events have still been reported (Ajithkumar et al. 2025; Ajithkumar et al. 2024), and their underlying causes require further investigation. In previous studies, we showed that distinct *Francisella* isolates were associated with variable virulence levels in mussels from the same batch (Bouras et al. 2024). As recently shown for the oyster pathogen *Vibrio aestuarianus*, understanding the genetics of the pathogen and its potential virulence markers is critical to anticipating disease and developing mitigation strategies (Mesnil et al. 2023).

From our previous study, the isolate FR21 showed noticeable differences from other *F. halioticida* isolates, both on the primary identification aspect (16s RNA, genotyping *rpoB*) (Bouras et al. 2023) and in virulence (Bouras et al. 2024). The major aim of this study was to obtain the phenotypic profiles of the FR22 isolates to support these preliminary observations. Here, FR21 remains distinct in several phenotypic aspects. In microscopy, FR22 isolates differ from FR21 in several ways. First, FR21 displays a shape ranging from cocci to bacilli, with a majority of coccobacilli when grown in MEB 2 mM FeCl_3_ (Bouras et al. 2023). In contrast, FR22 isolates lacked extracellular formations but consistently exhibited polar invaginations in both SEM and TEM, which were not observed in FR21. The polar invaginations, though seemingly minor, are persistent, which suggest a stable morphological marker within these strains and help exclude the possibility of a preparation artifact. However, confirmation via cryo‐fixation would help affirm the presence of this invagination. No similar invagination has been observed in the literature, though it is reminiscent of the planctomycete *Gemmata obscuriglobus* (Sagulenko et al. 2014) or the magnetotactic *Magnetospirillum* spp. (Barber‐Zucker et al. 2016), these traits can potentially be fixed and inherited. However, no potential function can be linked.

*F. halioticida* is known to exhibit resistance to several antibiotics' families, for example, ampicillin, erythromycin, cefuroxime, and polymyxin B (Brevik et al. 2011; Kawahara et al. 2019). While the combination of the previous isolation media seemed suitable for the isolation from frozen hemolymph of abalone (Kamaishi et al. 2010) or muscle abscess from Yesso scallops (Kawahara et al. 2019), this medium did not seem perfectly suited for mussel tissues (Bouras et al. 2023). In mussel farming, the use of antibiotics is nearly impossible to apply due to the low manutention of poles and the exploitation in natural environment. For this reason, testing the susceptibility of these isolates may not seem particularly pertinent. However, assessing resistance is useful for improving the antimicrobial cocktail used in the isolation media and for determining whether resistance profiles are consistent among isolates. When supplementing culture media with antibiotics, it is important to vary the class of antibiotics used. In this study, the isolates were tested individually using broth µ‐dilution to gain a clearer picture of their resistance patterns. The selected temperature, 17°C, reflects the temperature found optimal to isolate *F. halioticida*. While 15°C strongly delay the apparition of the first colony, 20°C resulted in excessively high contamination despite the use of three antibiotics. The conventional recommendation of the EUCAST relies on 15°C for psychrophilic organisms, 20°C, 22°C, or 25°C for aquatic pathogens. Given this context, we chose not to follow the EUCAST recommendations. This deviation, while precluding comparison with standard recommended values, was necessary to achieve the main goal of the study, improving the isolation medium.

In *Francisella*, antibiotic resistance does not appear to be plasmid‐borne, suggesting that horizontal gene transfer is unlikely (Challacombe, Petersen, et al. 2017; Kassinger and van Hoek 2021). In silico predictions of resistance markers across *Francisella* genomes have mostly highlighted the presence of multidrug transporters, which are encoded on the chromosome (Caspar and Maurin 2017; Kassinger and van Hoek 2021). If the resistance profile of *F. halioticida* is compared with CLSI guidelines for *F. tularensis* (Jorgensen et al. 2007), most isolates would be considered resistant to several antibiotics, including doxycycline, ciprofloxacin, gentamicin or streptomycin. The high resistance levels observed in *F. halioticida* are concerning. Marine environments are known reservoirs of resistance genes, and phenotypic resistance to various antibiotics is widespread among marine bacteria (Hatosy and Martiny 2015).

Conventional phenotypical differences can provide significant insights into bacterial classification, but modern sequencing techniques allow for more rapid and comprehensive classification and the identification of virulence markers (Palittapongarnpim 2024). The phenotypic findings from this study indicate that classification based solely on the 16S rRNA gene may be misleading, a limitation widely documented across bacterial taxa. To resolve this ambiguity, we sequenced and analyzed the complete circular genomes of all isolates obtained from mussels.

The increasing availability of bacterial genome sequences has facilitated taxonomic identification. Many species within or related to the genus *Francisella* have been described in the past two decades. In the early 2000s, two major fish pathogens have been described, *F. noatunensis* and *F. orientalis* affecting mainly the Atlantic cod *Gadus morhua* and the tilapia *Oreochromis* sp., respectively. Since then, numerous environmental *Francisella* species have also been described, sometimes without a clear association with a host (Challacombe, Petersen, et al. 2017; Kumar et al. 1928).

The presence of pseudogenes in our assemblies is consistent with other *Francisella* genomes, such as *F. orientalis* Toba04 (Sridhar et al. 2012). While some pseudogenes may impact virulence (Gunnell et al. 2016), others can still be expressed despite being mutated (Feng et al. 2022). Our hybrid assemblies yielded complete, circular chromosomes with high‐quality base calls. Multiple plasmids were observed in isolates obtained without freezing, suggesting a possible loss of plasmids during storage. These may be cryptic plasmids, as previously reported in environmental and pathogenic *Francisella* strains (Challacombe, Petersen, et al. 2017). Interestingly, pathogenic isolates had fewer coding sequences and more pseudogenes, a trend observed in other *Francisella* species (Rohmer et al. 2007; Sridhar et al. 2012)

The reference genome DSM23729 has previously been aligned with the Japanese isolate UTH170823 (Kawahara et al. 2021), revealing strong differences. Our analysis confirms these discrepancies even after genome rotation to a common starting gene (*dnaA*). Despite this, isolates from three different hosts (abalone, Yesso scallop, and mussel) and continents (Asia, North America, Europe), show highly conserved genomic organization, suggesting a stable pathogenic structure among virulent *F. halioticida* strains.

Regarding virulence, we confirmed the presence of genes involved in host cell entry (*DeoB, PilT/F*), phagosomal escape (*IglA‐D, MglA/FevR*, and *DsbA*), and intracellular replication (*iglD*, among others) (Meibom and Charbit 2010). The major virulence determinant in *Francisella* species, the Pathogenicity Island (FPI), was found to be present in all isolates except FR22b. The FPI was found to be flanked by transposases, which is common (Hacker and Kaper 2000), but those transposases belonged to two distinct families: IS630 for FR21 and IS3 for FR22, 8472‐13A, DSM23729, and UTH170823. The FPI's absence in FR22b can be explained by two scenarios: loss through recombination events in FR22b or acquisition by the common ancestor of the other isolates, followed by vertical inherence (Hacker and Kaper 2000; Schmidt and Hensel 2004). In *F. halioticida*, the FPI is located downstream of an rRNA operon and shows a conserved gene structure among virulent strains. The absence of a histidine kinase in 8472‐13A suggests a disruption of a two‐component regulatory system, which may influence its virulence. Finally, a pseudo‐FPI cluster was identified in AG1 but lacked key determinants, further supporting its taxonomic distinctiveness.

From a taxonomic standpoint, our results suggest FR21 warrants further analysis that could lead to its classification as a potential new subspecies. Although its ANI and dDDH values are superior to the conventional breakpoints (95% ANI and 70% dDDH) (Goris et al. 2007; Konstantinidis and Tiedje 2005a, 2005b), they remain low and close to this threshold. Isolate AG1, however, remains phylogenetically distant from known *Francisella* species. Its placement in the pan‐genomic tree alongside related genera and its genomic divergence strongly suggest it may represent a novel genus. Current debates surrounding the classification of *F. endociliophora* support a re‐examination of genus‐level boundaries within Francisellaceae (Kumar et al. 1928; Öhrman et al. 2021). Given that these observations rely heavily on ANI and dDDH analysis, further research should be performed to evaluate the reclassification of both isolates.

## 5 Conclusion

Altogether, our results reinforce the necessity for genomic approaches in pathogen identification and support the application of targeted molecular tools in aquaculture surveillance. The genomic diversity observed even within isolates from a single farming site underscores the complexity of francisellosis and highlights its implications for disease management.

## Author Contributions

**Helene Bouras:** conceptualization (equal), methodology (lead), validation (equal), formal analysis (lead), investigation (lead), data curation (equal), visualization (lead), writing – original draft (lead). **Yann Quesnelle:** methodology (supporting), investigation (supporting). **Suzanne Trancart:** methodology (supporting), investigation (supporting). **Didier Goux:** methodology (supporting), investigation (supporting). **Jean‐Louis Blin:** resources (lead), project administration (supporting). **Manuel Savary:** project administration (supporting), funding acquisition (lead). **Maryline Houssin:** conceptualization (equal), methodology (supporting), validation (equal), project administration (supporting), funding acquisition (supporting), supervision (equal), writing – review and editing (supporting). **Celine Zatylny‐Gaudin:** conceptualization (equal), methodology (supporting), validation (equal), project administration (supporting), funding acquisition (supporting), supervision (equal), writing – review and editing (lead).

## Ethics Statement

The authors have nothing to report.

## Conflicts of Interest

The authors declare no conflicts of interest.

## Supporting information


**Supplementary Figure 1:** Scanning electron microscopy observations of *Francisella halioticida* isolates of the FR22 type. **Supplementary Figure 2:** Transmission microscopy observations of Francisella halioticida isolates of FR22 type. **Supplementary Table 1:** List of genomes used in this study. **Supplementary Table 2:** Antibiotics used to determine the minimum inhibitory concentration of *Francisella* sp. isolates PBtp = 0.1 M phosphate buffer pH 8 – DMSO : Dimethylsulfoxide. **Supplementary Table 3:** Phenotypic characterization of *Francisella* spp. isolates obtained from mussels found on the French coast. **Supplementary Table 4:** Accession numbers for Biosample, raw reads obtained from Illumina (short‐reads) and PacBio (long‐read) sequencing, as well as the chromosomal assembly.

## Data Availability

Raw data and assembly of the isolates FR22a, FR22b, FR22c, FR22d, FR21, AG1, and 8472‐13A are available in GenBank within the BioProject PRJNA1275855. All accession numbers are available in Table S4.
