# Supplementary material for: Phenotypical and Genomic Characterization of the Mollusk Pathogen Francisella halioticida
Source: Microbiologyopen. 2025 Nov 28;14(6):e70172. doi: 10.1002/mbo3.70172 (PMC12661370; doi:10.1002/mbo3.70172)
Supplement: Supplementary file 1 — Supplementary Figure 1: Scanning electron microscopy observations of Francisella halioticida isolates of the FR22 type. Supplementary Figure 2: Transmission microscopy observations of Francisella halioticida isolates of FR22 type. Supplementary Table 1: List of genomes used in this study. Supplementary Table 2: Antibiotics used to determine the minimum inhibitory concentration of Francisella sp. isolates PBtp = 0.1 M phosphate buffer pH 8 – DMSO : Dimethylsulfoxide. Supplementary Table 3: Phenotypic characterization of Francisella spp. isolates obtained from mussels found on the French coast. Supplementary Table 4: Accession numbers for Biosample, raw reads obtained from Illumina (short‐reads) and PacBio (long‐read) sequencing, as well as the chromosomal assembly. [file MBO3-14-e70172-s001.docx]

**Supplementary Table 1. List of genomes used in this study.** The species, strain, Genbank accession number and assembly accession number are presented for all strains.

| Species | Strain | N° Accession | Assembly |
| --- | --- | --- | --- |
| *Francisella tularensis* subsp. *tularensis* | SCHU S4 | CP010290.1 | GCA_000833535.1 |
| *Francisella tularensis* subsp. *holarctica* | LVS | AM233362.1 | GCA_000009245.1 |
| *Francisella tularensis* subsp. *mediasiatica* | 2023/540 | CP130001.1 | GCA_030505515.1 |
| *Francisella tularensis* subsp. *novicida* | Fx1 | CP002557.1 | GCA_000195535.1 |
| *Francisella tularensis* subsp. *novicida* | U112 | CP009633.1 | GCA_000833375.1 |
| *Francisella orientalis* | F1 | CP018051.1 | GCA_001885275.1 |
| *Francisella orientalis* | Toba 04 | CP003402.1 | GCA_000262205.1 |
| *Francisella noatunensis* subsp. *noatunensis* | FSC774 | CP053850.1 | GCA_014844275.1 |
| *Francisella philomiragia* | ATTC 25015 O319L | CP010019.1 | GCA_000833455.1 |
| *Francisella philomiragia* | FSC153 | CP009442.1 | GCA_000833295.1 |
| *Francisella uliginis* | TX07-7310 | CP016796.1 | GCA_001895265.1 |
| *Francisella salimarina* | E95-16 | CP043552.1 | GCA_008369785.1 |
| *Francisella salimarina* | CHUGA-F75 | CP076680.1 | GCA_018972105.1 |
| *Francisella persica* | ATCC VR-331 | CP013022.1 | GCA_001653955.1 |
| *Francisella opportunistica* | 14-2155 | CP022375.1 | GCA_003347095.1 |
| *Francisella opportunistica* | MA067296 | CP016930.1 | GCA_001879645.1 |
| *Francisella hispaniensis* | FSC454 | CP018093.1 | GCA_001885235.1 |
| *Francisella* sp. | LA112445 | CP041030.1 | GCA_012224145.1 |
| *Allofrancisella guangzhouensis* | 08HL01032 | CP010427.1 | GCA_000815225.1 |
| *Allofrancisella frigidaquae* | SYSU 10HL1970 | CP038017.1 | GCA_012222825.1 |
| *Pseudofrancisella aestuarii* | SYSU WZ-2 | ASM357447.2 | GCA_003574475.2 |
| *Pseudofrancisella frigiditurris* | CA97-1460 | CP009654.1 | GCA_001880225.1 |
| *Parafrancisella adeliensis* | FDC440 | CP021781.1 | GCA_003290445.1 |
| *Fangia hongkongensis* | DSM21703 | ASM37944v1 | GCF_000379445.1 |

**Supplementary Table 2. Antibiotics used to determine the minimum inhibitory concentration of *Francisella* sp. isolates PB 0.1 M phosphate buffer pH 8 – DMSO : Dimethylsulfoxide**

| Family | Acronym | Antibacterial agent | Supplier | Ref | Conc. max | Solvent |
| --- | --- | --- | --- | --- | --- | --- |
| β-lactams/Penicillins | AMP | Ampicillin | SIGMA | A5354 | 512 µg/mL | PB |
|  | PEN | Penicillin | TCI | P1770 | 512 µg/mL | H2O |
|  | AMX | Amoxicillin | Acros | 455140050 | 512 µg/mL | PB |
| β-lactams/Cephalosporines | CXM | Cefuroxime | MCE | HY-B1256 | 512 µg/mL | H2O |
| Aminosides/Aminoglycosides | STR | Streptomycin | SIGMA | 46754 | 128 µg/mL | H2O |
|  | TOB | Tobramycin | SIGMA | T4014 | 32 µg/mL | H2O |
|  | GEN | Gentamicin | SIGMA | G3632 | 32 µg/mL | H2O |
|  | KAN | Kanamycin | Applichem | A4718 | 512µg/mL | H2O |
| (Fluoro)Quinolones | CIP | Ciprofloxacin | FLUKA | 33434 | 16 µg/mL | H2O |
|  |  | Enrofloxacin | SIGMA | 17849 | 16 µg/mL | 0,01 M HCl |
|  |  | Sarafloxacin | FLUKA | 33497 | 16 µg/mL | 0,2 M HCl |
|  |  | Flumequine | SIGMA | 45735 | 32 µg/mL | DMSO |
|  | NAL | Nalidaxic acid | SIGMA | N8878 | 16 µg/mL | 0,5 M NaOH |
| Macrolides | ERY | Erythromycin | SIGMA | E5389 | 128 µg/mL | 100% EtOH |
|  |  | Natamycin | FLUKA | R1116 | 64 µg/mL | DMSO |
|  | AMB | Amphotericin B | Applichem | A1907 | 32 µg/mL | DMF |
| Tetracyclines |  | Oxytetracycline | Biokar | 6B50080 | 8 µg/mL | H2O |
|  | DOX | Doxycycline | SIGMA | D9891 | 16 µg/mL | H2O |
| Polypeptides | PMB | Polymixin B | Pan Reac | A0890 | 128 µg/mL | H2O |
| Sulfamides | SMX | Sulfamethoxazole | HPC | 674029 | 512 µg/mL | DMSO |
| Diaminopyrimidines | TMP | Trimethoprim | HPC | 672980 | 32 µg/mL | 0,05 M HCl |
| Phenicols |  | Florfenicol | HPC | 674759 | 256 µg/mL | 100% EtOH |
| Rifamycin | RIF | Rifampicin | FLUKA | 46713 | 32 µg/mL | Methanol |
| Fungicide |  | Cycloheximide | SERVA | 1070004 | 64 µg/mL | H2O |

**Supplementary Table 3. Phenotypic characterization of *Francisella* spp. isolates obtained from mussels found on the French coast.** The isolates were seeded on API 32A, 32E, 20E and ZYM media. These media were incubated for 24 hours at 20°C. Reactions showing differences between *Francisella halioticida* isolates are highlighted. W: weak signal, NR: not realized

| Enzymes/Réactions | 8472-13A | FR21 | FR22a | FR22b | FR22c | FR22d | AG1 | AG3 |
| --- | --- | --- | --- | --- | --- | --- | --- | --- |
| Esterase (C4)/Esterase lipase (C8) | + | + | W | W | W | W | NR | NR |
| Lipase (C14) | - | - | - | - | - | - | NR | NR |
| Trypsin, α-chymotrypsin | - | - | - | - | - | - | NR | NR |
| Alkaline phosphatase | W | W | W | W | W | W | W | - |
| Acid phosphatase | + | + | + | + | + | + | NR | NR |
| Naphthol-AS-Bi-phosphohydrolase | + | W | W | W | W | W | NR | NR |
| α-galactosidase, α-glucosidase, α-mannosidase, α-fucosidase, α-arabinosidase, β-galactosidase, β-galactosidase-6-P, β-glucosidase, N-acetyl-β-glucosaminidase | - | - | - | - | - | - | - | - |
| β-glucuronidase | - | W | - | - | - | - | - | - |
| O-Nitrophenyl N-acetyl Beta-glucosaminide, P-Nitrophenyl - beta g galactopyranoside | - | - | - | - | - | - | - | - |
| Urease, Nitrate reduction, Indole test | - | - | - | - | - | - | - | - |
| Acetoin production | - | + | - | - | - | - | - | - |
| Prolyl aminopeptidase | + | + | + | + | + | + | - | - |
| Leucyl glycine aminopeptidase | - | - | - | - | - | - | - | + |
| Histidine, Glutamyl glutamic acid, cystine aminopeptidase | - | - | - | - | - | - | - | - |
| Phenylalanine aminopeptidase | W | + | - | - | - | - | - | - |
| Leucine aminopeptidase | + | + | + | + | + | W | + | - |
| Pyroglutamic acid aminopeptidase | + | + | + | + | + | + | - | - |
| Glycine, alanine aminopeptidase | + | + | + | + | + | + | + | + |
| Serine aminopeptidase | + | + | W | W | + | W | + | - |
| Arginine aminopeptidase | + | + | - | - | - | W | - | - |
| Tyrosine aminopeptidase | - | + | - | - | - | - | W | - |
| Valine aminopeptidase | - | - | - | - | - | - | NR | NR |
| Lysine, Ornithine, glutamic acid decarboxylase | - | - | - | - | - | - | - | - |
| Aesculin | - | - | - | - | - | - | - | - |
| D-glucose, L-arabinose, adonitol, L-rhamnose, D-mannitol, D-sorbitol, D-cellobiose, D-melibiose, sodium glucuronate, D-maltose, D-trehalose, sucrose/saccharose, 5-ketogluconate, palatinose, galacturonic acid, colistin sulfate, p-coumaric acid raffinose fermentation/oxidation | - | - | - | - | - | - | - | - |
| D-Mannose fermentation | - | + | - | - | - | - | W | - |
| Phenylalanine desaminase, Tetrathionate reductase | - | - | - | - | - | - | - | - |
| Indoxyl phosphate | W | W | W | W | W | W | + | - |

**Supplementary Table 4. Accession numbers for Biosample, raw reads obtained from Illumina (short-reads) and PacBio (long-read) sequencing, as well as the chromosomal assembly. All data are gathered within the Bioproject PRJNA1275855. NR : non relevant.**

| Isolate | BioSample | Raw reads – Illumina | Raw reads - Pacbio | Assembly |
| --- | --- | --- | --- | --- |
| FR21 | SAMN49024706 | SRR34104085 | SRR34104084 | JBSFBD000000000 |
| FR22a | SAMN49024702 | SRR34104095 | SRR34104094 | JBSFAZ000000000 |
| FR22b | SAMN49024703 | SRR34104091 | SRR34104090 | JBSFBA000000000 |
| FR22c | SAMN49024704 | SRR34104089 | SRR34104088 | JBSFBB000000000 |
| FR22d | SAMN49024705 | SRR34104087 | SRR34104086 | JBSFBC000000000 |
| 8472-13A | SAMN49024707 | SRR34104093 | SRR34104092 | JBSFBE000000000 |
| AG1 | SAMN52039301 | NR | SRR35685434 | JBSHYB000000000 |


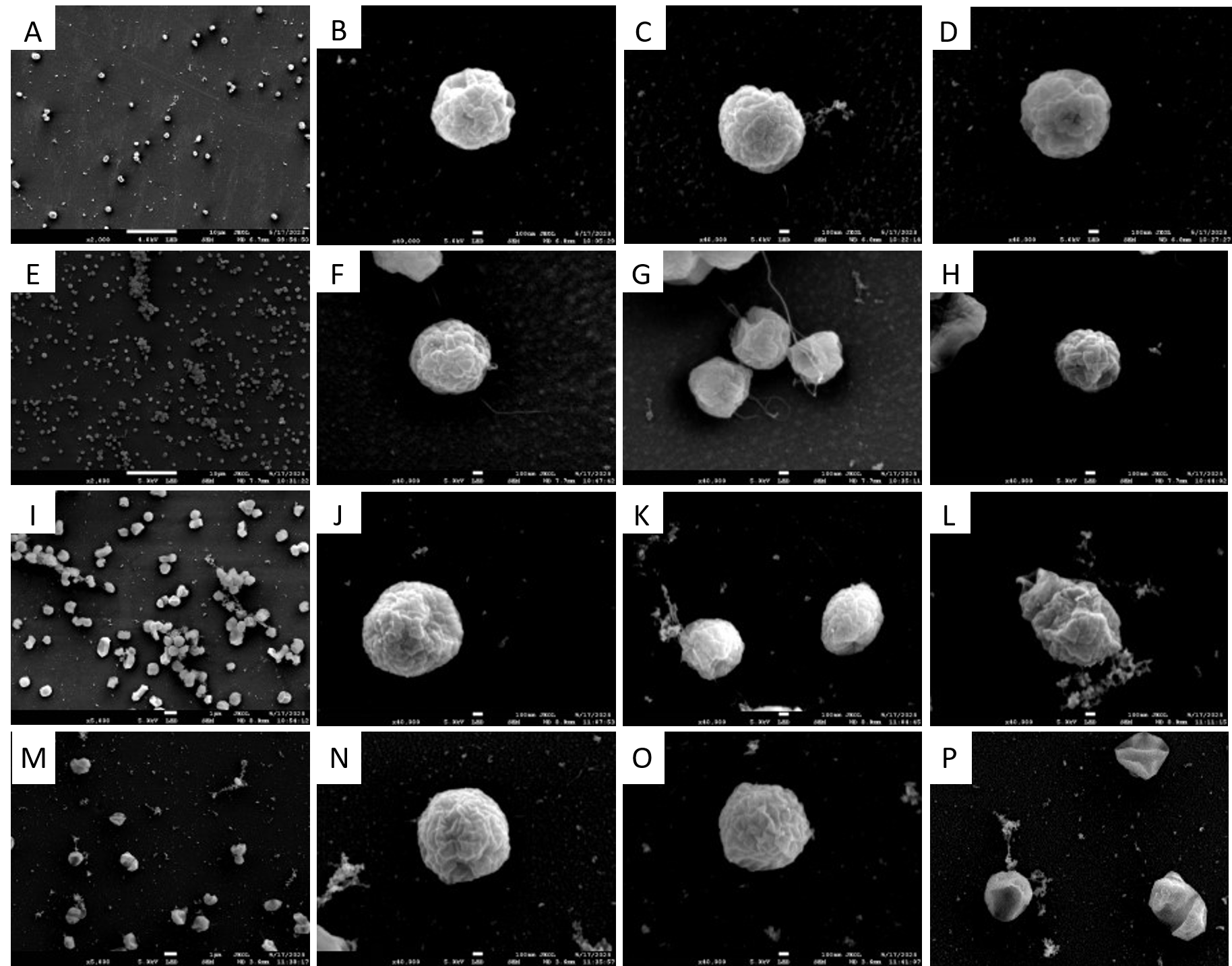


**Supplementary Figure 1. Scanning electron microscopy observations of *Francisella halioticida* isolates of the FR22 type.** A-D: FR22a, E-H: FR22b, I-L: FR22c, M-P: FR22d. Scale A, E: 10 µm. Scale I, M: 1 µm. Scale B, C, D, F, H, J, K, L, N, O, P: 100 nm.


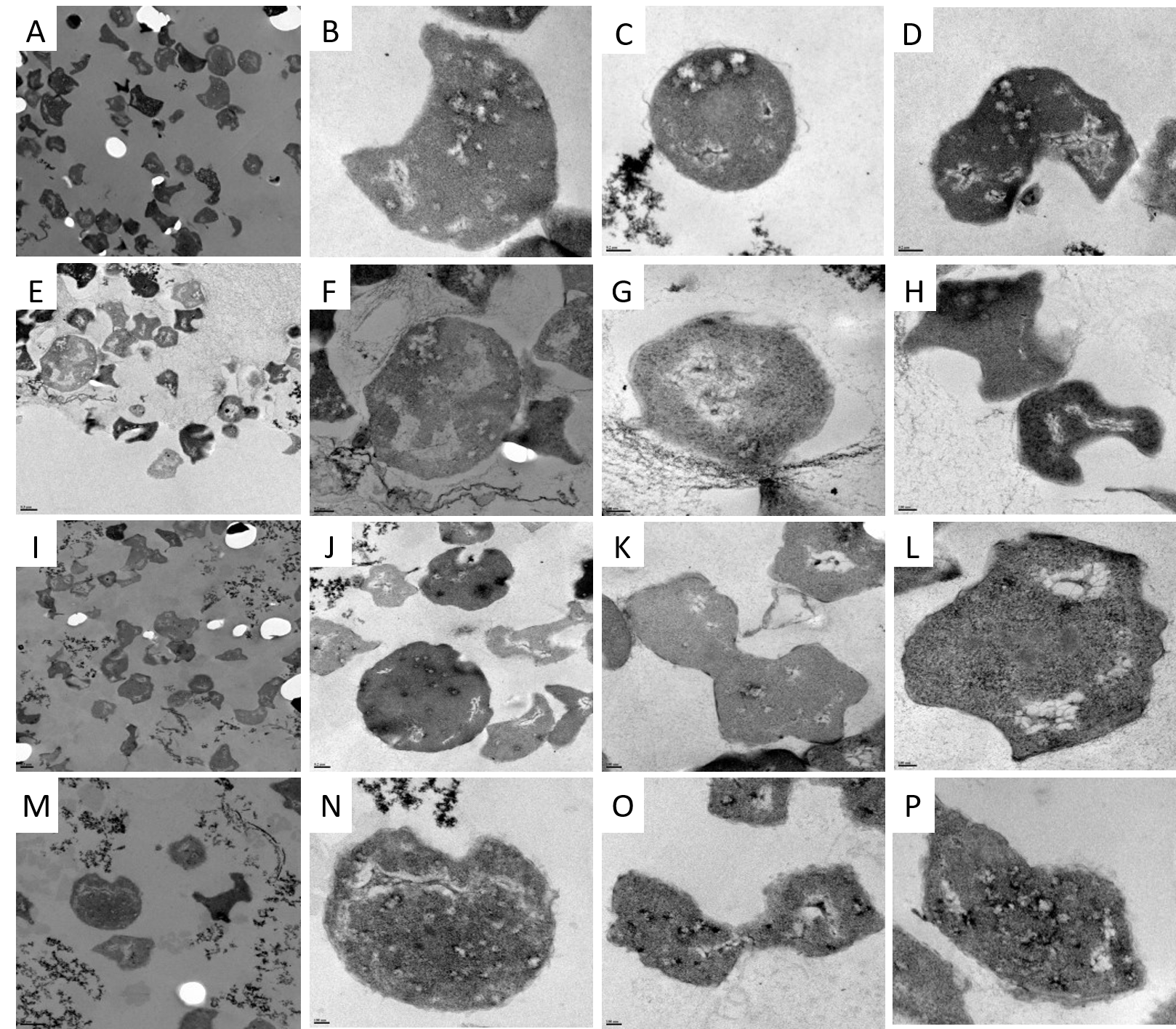


**Supplementary Figure 2. Transmission microscopy observations of Francisella halioticida isolates of FR22 type.** A-D: FR22a, E-H: FR22b, I-L: FR22c, M-P: FR22d. Scale A, E, I, M: 0.5 µm. Scale B, C, D, F, J: 0.2 µM. Scale G, H, K, L, N, O, P: 100 nm.
